# Predictors of recurrence of atrial tachyarrhythmias after pulmonary vein isolation by functional and structural mapping of nonparoxysmal atrial fibrillation

**DOI:** 10.1002/joa3.12670

**Published:** 2021-12-23

**Authors:** Koji Kumagai, Tsukasa Sato, Yuki Kurose, Takenori Sumiyoshi, Kaoru Hasegawa, Yuko Sekiguchi, Minoru Yambe, Tatsuya Komaru

**Affiliations:** ^1^ Department of Cardiovascular Medicine Tohoku Medical and Pharmaceutical University Miyagi Japan

**Keywords:** atrial fibrillation, dominant frequencies, low‐voltage areas, pulmonary vein isolation, rotors

## Abstract

**Background:**

This study aimed to evaluate the predictors of recurrence of atrial tachyarrhythmias by structural and functional mapping: voltage, dominant frequency (DF), and rotor mapping after a pulmonary vein isolation (PVI) in nonparoxysmal atrial fibrillation (AF) patients.

**Methods:**

A total of 66 nonparoxysmal AF patients were prospectively investigated. After the PVI, an online real‐time phase mapping system was used to detect the location of rotors with critical nonpassively activated ratios (%NPs) of ≧50% in each left atrial (LA) segment, and high‐DFs of ≧7 Hz were simultaneously mapped. After restoring sinus rhythm, low‐voltage areas (LVAs < 0.5 mV) were mapped using the Advisor HD grid catheter (HDG).

**Results:**

Sixty‐four of 66 (97%) AF patients had minimum to mild LVAs regardless of an enlarged LAD and LA volume (45 ± 6.0 mm and 141 ± 29 ml). There were no significant differences in the max and mean DF values and %NPs between the patients with and without recurrent atrial tachyarrhythmias. However, there was a significant difference in the LVA/LA surface area between the patients with and without recurrent atrial tachyarrhythmias (*p *= .004). Atrial tachyarrhythmia freedom was significantly greater in those with LVAs of ≤3.3% than in those >3.3% after one procedure over 11.6 ± 0.8 months of follow‐up (77.1% vs. 33.3%, *p *< .001). In a multivariate analysis, the LVA/LA surface area after the PVI (HR 1.079; CI, 1.025–1.135, *p *= .003) was an independent predictor of AF recurrence.

**Conclusions:**

The predictor of atrial tachyarrhythmia recurrence after the PVI was LVAs rather than DFs and rotors in nonparoxysmal AF patients.

## Graphical abstract

The LVAs were an independent predictor of recurrent AF/AT after the PVI. The HDG might exclude false LVAs, which would help detect the AF substrate more accurately. LVAs detected accurately by the HDG might be more selective targets than DFs and rotors alone after the PVI in nonparoxysmal AF patients with atrial remodeling.

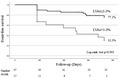

## 1 INTRODUCTION

Pulmonary vein isolation (PVI) has been the cornerstone of atrial fibrillation (AF) ablation. However, the single procedure success rates are limited, particularly in persistent and long‐standing persistent AF.^1^ Additional strategies including linear ablation and ablation of complex fractionated atrial electrograms (CFAEs) ^2^, ^3^ have not indicated any efficacy benefit over a PVI alone in nonparoxysmal AF patients.^4^

A strategy based on low‐voltage areas (LVAs) as detected by left atrial (LA) structural voltage mapping during sinus rhythm (SR) has recently been reported because LVAs are a predictor of AF recurrence after AF ablation.^5^, ^6^, ^7^ The recent utility of the Advisor HD grid (HDG) mapping catheter (Abbott Technologies) has been reported to lead to smaller LVAs as compared to a conventional mapping catheter because it contributes to the bipolar recording regardless of the direction of the activation.^8^

Furthermore, functional mapping as automatic detection of high‐DF sites and rotational activations by a novel phase mapping system may potentially selectively target localized sources maintaining AF in nonparoxysmal AF patients.^9^, ^10^, ^11^, ^12^, ^13^ We previously reported the importance of high‐DF sites overlapping with LVAs that are present using a conventional mapping catheter after the PVI.^14^ However, the predictors of recurrence of atrial tachyarrhythmias detected after the PVI by both structural and functional mapping of nonparoxysmal AF remain unclear. This study aimed to evaluate the predictors of recurrence of atrial tachyarrhythmias by evaluating the LVAs, DFs, and rotors after the PVI in nonparoxysmal AF patients.

## 2 METHODS

### 2.1 Study population

The present study was a prospective observational study that included 74 consecutive patients with nonparoxysmal AF who underwent catheter ablation at our institution between February 25, 2019 and April 2, 2020. Five patients were excluded due to having no multidetector computed tomography (MDCT) images and three for undergoing additional LA ablation other than the PVI. Finally, a total of 66 persistent and long‐standing persistent AF patients were investigated in this study. Persistent AF was defined as AF lasting ≥7 days but <1 year, and long‐standing persistent AF as continuous AF lasting ≥1 year.^15^ All anti‐arrhythmic drugs (AADs) were discontinued for at least 5 half‐lives and one patient received oral amiodarone therapy before the catheter ablation. The protocol for this research project was approved by a suitably constituted Ethics Committee of Tohoku Medical and Pharmaceutical University (Date of IRB approval: February 25, 2019; Approval number, 2018‐2‐104) and it conformed to the provisions of the Declaration of Helsinki. All patients provided written informed consent for the ablation procedure, and the use of their anonymized data in this study.

### 2.2 Catheter ablation procedure

The catheter ablation procedure was performed using a NavX system (St. Jude Medical) as described previously.^12^, ^14^, ^16^ A 5‐french deflectable catheter was inserted into the coronary sinus (CS) via the right femoral vein. After a single transseptal procedure under intracardiac echocardiography guidance, an 8‐F SL0 sheath and Agilis sheath (St. Jude Medical) were advanced into the LA. After the transseptal procedure, a single bolus of 5000 U of heparin was administered. A continuous infusion with heparinized saline was delivered to maintain an activated clotting time of 300 to 350s. The 3D LA geometry was created using a 7‐F decapolar circular catheter (EPstar Libero, Japan Lifeline Co., Ltd.). The whole LA was divided into eight areas (PVs, roof, left atrial appendage [LAA], septum, and lateral, anterior, inferior, and posterior regions) for a location analysis of the LVAs, DFs, and rotors.^12^, ^14^, ^16^ The mapping points in each region were similar in number and nearly equally distributed (LA mean mapping points: 1843 ± 501).

The PVI was performed guided by a 7‐F decapolar circular catheter positioned at the PV ostia as described previously.^12^, ^14^, ^16^ Each radiofrequency (RF) energy application was delivered for 40s. A 3.5 mm irrigated tip RF catheter (FlexAbility^TM^, St. Jude Medical Inc.) was used with the temperature limited to 42℃ and power to 30W (25W for sites near the esophagus) with a flow rate of 13 ml/min. After the PVI, DF mapping and rotor mapping were simultaneously performed in the same mapping area using a deflectable 20‐pole spiral‐shaped catheter with a diameter of 2.5 cm (Reflexion HDTM, St. Jude Medical). Finally, a LA voltage map was performed using the HDG during pacing from the distal CS after external cardioversion.

Once in SR, decremental pacing (10 ms steps from 250 to 200 ms, over a period of 10 s) at an output of 10 mA and 2 ms pulse width was performed from the distal CS once, in an attempt to induce an atrial tachyarrhythmia without an isoproterenol injection. An induced AF/AT (atrial tachyarrhythmias) was defined as that sustained for at least 2 min.^15^ When AF/AT continued, external cardioversion was performed. When cavotricuspid isthmus (CTI)‐dependent AFL was induced, a CTI ablation was performed. The endpoint of this study was AF/AT recurrence.

### 2.3 Frequencies analysis

After the PVI, DF mapping during AF in each area was performed using the fast Fourier transform (FFT) method described previously.^12^, ^14^, ^16^ Recordings at each site for 5 s were performed using a deflectable 20‐pole spiral‐shaped catheter with a diameter of 2.5 cm (Reflexion HDTM, St. Jude Medical). Signals were truncated to 5 s at a sampling rate of 1000 Hz, providing 4096 points for analysis (resolution 0.50 Hz). The signals were rectified and processed by a Hanning window function and filtered from 2 to 20 Hz. The DF analysis was performed by an offline FFT analysis using the software implemented in the polygraph (RMC‐5000; Nihon Kohden Co.) in real time and then the DF values were input manually into the NavX system. The DF value was determined as the frequency associated with the maximum peak power of the spectrum. Only DF points with a regularity index ≥0.2 were included.^12^, ^14^, ^16^ The high‐DF sites were defined as DFs of ≥7 Hz. The highest value of the DF in each mapping area in the LA was measured and calculated.

### 2.4 Real‐time phase mapping

In the same areas where the DF mapping was performed, mapping during AF was simultaneously performed using an online real‐time phase mapping system (ExTRa MappingTM, Nihon Kohden Co.) as described previously.^17^ This mapping system was based on 41 bipolar intra‐atrial electrograms (including nine virtual electrograms) recorded by a deflectable 20‐pole spiral‐shaped catheter with a diameter of 2.5 cm (Reflexion HDTM, St. Jude Medical). The contact was confirmed by the recorded electrograms, fluoroscopy, and 3D geometry. The distance between a mapping point and the geometry surface created by the EnSite NavX was set at 5 mm. The data sampling was adopted as good contact in areas where sufficient electrograms could be recorded from the vast majority of the electrodes. Based on the 5‐s wave dynamics during AF, each phase map was automatically created by the ExTRa Mapping. Nonpassively activated areas, in which rotational activations were frequently observed, were automatically detected by the ExTRa Mapping. The value of the “nonpassively activated ratio” (%NP), which was the ratio of the form of the rotors and multiple wavelets that were assumed to contain AF drivers to the recording time, was automatically calculated from the 5‐s real‐time phase map created by the ExTRa Mapping.^17^ Of the 5‐s maps, the activation sequences during 720 ms (60 ms × 12 consecutive time windows) of representative episodes were depicted as images.

### 2.5 LVA mapping and analysis

After external cardioversion, a detailed bipolar LA voltage map was constructed during pacing from the distal CS in all patients. The LVA mapping method has been described previously.^5^, ^6^, ^7^ The mapping points were systematically acquired with the HDG, which has 16 electrodes and a 3 mm equidistant electrode spacing to create a high‐density contact voltage map via the Ensite Velocity 3D mapping system. The algorithm displayed the signals amalgamated from orthogonal recordings of each bipole and displayed the highest amplitude signal (HD wave solution). An interpolation threshold of 10 mm on the NavX system was used for the surface color projection. Adequate endocardial contact was evaluated by stable electrograms and consideration of the distance to the geometry surface. Only true sinus beats were selected. Bipolar electrograms were filtered by a bandpass of frequencies between 30 and 500 Hz. In accordance with the previous studies,^5^, ^6^, ^7^ an LVA was defined as an area with a bipolar peak‐to‐peak electrogram amplitude of <0.5 mV and electrical scar areas as <0.1 mV. The LA surface area was defined as the LA body area without the PV antrum regions inside the PVI line. The registration for evaluating the MDCT image with the NavX map consisted of an AF image imported (preablation) with a post cardioversion SR map in all patients in order to obtain the anatomical information, and the overlap between the LVAs and high‐DF sites was evaluated manually by two independent blinded observers.

### 2.6 Postprocedure care and follow‐up

Anti‐arrhythmic medications were continued for at least 3 months to prevent any early recurrences. A clinical interview, surface ECG, and 24‐h Holter monitoring were performed 1 day after the procedure and repeated 1, 3, 6, and 12 months after the catheter ablation. AF/AT recurrence was defined as sustained AF/AT lasting more than 30 s, which occurred more than 3 months after the catheter ablation.^15^

### 2.7 Statistical analysis

The continuous variables are presented as the mean ± standard deviation together with the 95% confidence intervals or median with interquartile ranges (IQR) (25th and 75th percentile). The correlation of the LVA/LA surface between that using two different HDG bipolar configurations was analyzed by a Spearman correlation analysis. Categorical variables were expressed as numbers and percentages. The significance of any differences between the two groups was analyzed with an unpaired *t*‐test and Mann‐Whitney U test for continuous variables, and with a Fisher's exact probability test for categorical variables. A predictive analysis of AF recurrence during the follow‐up period was assessed using multivariate Cox proportional hazard regression models. A multivariate analysis with multivariate Cox proportional hazard regression models was performed to isolate the independent criteria of AF recurrence after ablation. Only the variables with significant *p*‐values in the univariate analysis were included in the multivariate Cox proportional hazard regression analysis. A Kaplan‐Meier event‐free survival analysis was conducted to assess the cumulative freedom from AF recurrence. A value of *p* < .05 was considered statistically significant.

## 3 RESULTS

### 3.1 Patient characteristics

The AF patients were divided into two groups: patients without AF/AT recurrences (*n* = 43) and those with AF/AT recurrences (*n* = 23). One patient had AT recurrences during the follow‐up period. The patient characteristics are shown in Table 1. The patient characteristics and laboratory data except for the LV ejection fraction did not significantly differ between the two groups. The number of failed AADs were a mean of 0.2 ± 0.4. None except for six patients (two with hypertrophic cardiomyopathy, three with mitral regurgitation, and one with old myocardial infarctions) had structural heart disease.

**TABLE 1:** Patient characteristics

	All (*N* = 66)	No recurred AF (*N* = 43)	Recurred AF (*N* = 23)	*p*‐value
Age, years	65 ± 8	66 ± 8	63 ± 8	.164
Men, *n* (%)	51 (77)	33 (77)	18 (78)	.889
Duration of AF, mo	24 ± 18	23 ± 16	27 ± 20	.454
Persistent AF, *n* (%)	16 (24)	10 (23)	6 (26)	.798
Long‐standing persistent AF, *n* (%)	50 (76)	33 (77)	17 (74)	.798
CHA2DS2‐VASc score	2.1 ± 1.3	2.1 ± 1.3	2.0 ± 1.3	.835
Congestive heart failure, *n* (%)	12 (18)	10 (23)	2 (9)	.144
Hypertension, *n* (%)	41 (62)	26 (60)	15 (65)	.705
Diabetes mellitus, *n* (%)	15 (23)	7 (16)	8 (35)	.087
Prior stroke or TIA	4 (6)	3 (7)	1 (4)	.670
Structural heart disease, *n* (%)	5 (8)	3 (7)	2 (9)	.801
Body mass index	25 ± 4.3	25 ± 3.1	27 ± 5.6	.052
LA diameter (mm)	45 ± 6.0	44 ± 5.8	46 ± 6.2	.144
LVEF (%)	58 ± 8.6	57 ± 9.3	61 ± 6.2	.023
LA volume (ml)	141 ± 29	137 ± 30	148 ± 26	.129
BNP (pg/ml)	123 ± 81	128 ± 83	115 ± 79	.549
eGFR (ml/m/1.73 m^2^)	63 ± 16	64 ± 16	60 ± 17	.380

Abbreviations: AF, atrial fibrillation; BNP, B‐type Natriuretic Peptide; eGFR, estimated glomerular filtration rate; LA, left atrium; LVEF, left ventricular ejection fraction; RA, right atrium.

### 3.2 Procedural characteristics

The procedural characteristics are shown in Table 2. The total procedure time and the RF time for the PVI did not significantly differ between the two groups. There were no patients with AF termination. Therefore, all patients finally needed external cardioversion after the PVI.

**TABLE 2:** Procedural characteristics

	All (*N *= 66)	No recurred AF (*N* = 43)	Recurred AF (*N* = 23)	*p*‐value
Max DF value in LA, Hz	6.9 ± 0.7	6.8 ± 0.8	6.9 ± 0.8	.772
Max %NP, %	65.0 ± 11	64.4 ± 12	66.2 ± 9.8	.501
Mean DF value in LA, Hz	6.2 ± 0.7	6.2 ± 0.7	6.2 ± 0.7	.903
Mean %NP, %	38.0 ± 8.7	37.4 ± 8.6	39.4 ± 8.9	.384
high‐DFs ≧ 7 Hz, *n* (%)	36 (49)	22 (48)	14 (52)	.450
%NP ≧ 50%, *n* (%)	60 (91)	39 (91)	21 (91)	.935
high‐DFs ≧7 Hz with %NP ≧50%, *n* (%)	24 (34)	15 (33)	9 (37)	.733
high‐DFs ≧ 7 Hz with %NP < 50%, *n* (%)	29 (44)	18 (42)	11 (48)	.642
high‐DFs <7 Hz with %NP ≧50%, *n* (%)	52 (79)	34 (79)	18 (78)	.939
high‐DFs <7 Hz with %NP < 50%, *n* (%)	61 (92)	40 (93)	21 (91)	.801
LVAs/LA surface area after PVI, median (Q1–Q3), % (HD wave solution)	1.9 (0.9–3.5)	1.8 (0.8–2.5)	3.4 (1.0–5.6)	.004
LVAs/LA surface area after PVI, median (Q1–Q3), % (along spine)	4.6 (2.8–8.1)	4.0 (2.4–6.4)	7.4 (3.7–11.6)	.009
%NP ≧ 50% sites overlapped with LVAs, *n* (%)	23 (35)	13 (39)	10 (43)	.282
high‐DFs ≧ 7 Hz sites overlapped with LVAs, *n* (%)	13 (20)	5 (12)	8 (35)	.024
Cavotricuspid isthmus line	11 (17)	7 (16)	4 (17)	.908
Inducibility of AF after PVI, *n* (%)	7 (11)	4 (9)	3 (13)	.638
Total procedure time, min	192 ± 34	189 ± 40	195 ± 27	.561
RF time for PVI, min	30 ± 11	29 ± 10	31 ± 13	.547

Abbreviations: %NP, nonpassively activated ratio; AF, atrial fibrillation; DF, dominant frequency; LVAs, low‐voltage areas; PVI, pulmonary vein isolation; RA, right atrium; SR, sinus rhythm.

### 3.3 Frequencies and phase mapping analysis

**FIGURE 1:**
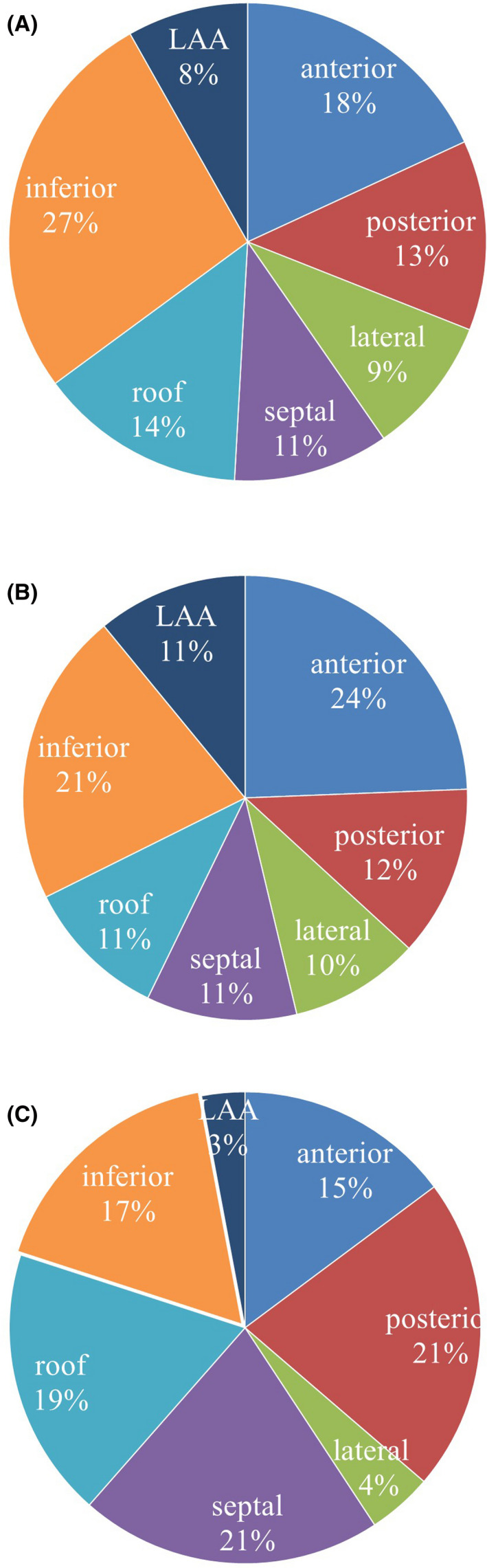
The distribution of the dominant frequency (DF)s, rotors, and low‐voltage areas (LVAs) in the left atrial (LA) after the pulmonary vein isolation (PVI). (A) The high‐DF sites ≧7 Hz were frequently identified in the inferior and anterior regions of the LA. (B) The %NPs ≧50% were frequently identified in the anterior and inferior regions of the LA. (C) LVAs were found at 135 sites in all patients. LVAs were frequently identified in the septal, posterior, and inferior regions of the LA

According to the frequencies and phase mapping analysis, all mapping sites (*n* = 761, 11.5 sites per patient) were divided into four types: high‐DFs ≧7 Hz with a %NP ≧50%, high‐DFs ≧7 Hz with a %NP <50%, high‐DFs <7 Hz with a %NP ≧50%, and high‐DFs <7 Hz with a %NP <50% (Table 3). However, there were no significant differences in the four types between the patients with and without recurrent AF/AT (Table 2). The max‐DF value and max‐%NP per patient were 6.9 ± 0.9 Hz and 65% ± 11% after the PVI, respectively. There were no significant differences between the patients with and without recurrent AF/AT in the max‐DF value, max‐%NP, mean DF value, and mean %NP (Table 2). The number of high‐DF sites of ≧7 Hz in the LA was 2.6 per patient after the PVI, and the number of %NPs of ≧50% was 8.9 per patient after the PVI. The distribution of the DFs, rotors, and LVAs in the LA after the PVI is indicated in Figure 1.

**TABLE 3:** Frequencies and phase mapping analysis

	DF ≧ 7 Hz	DF < 7 Hz	Total
%NP ≧ 50%, *n* (%)	75 (9.9)	126 (16.6)	201 (26.5)
%NP < 50%, *n* (%)	96 (12.7)	462 (60.9)	558 (73.5)
Total	171 (22.5)	588 (77.5)	759 (100)

Abbreviations: %NP, nonpassively activated ratio; DF, dominant frequency.

### 3.4 LVA mapping and analysis

**FIGURE 2:**
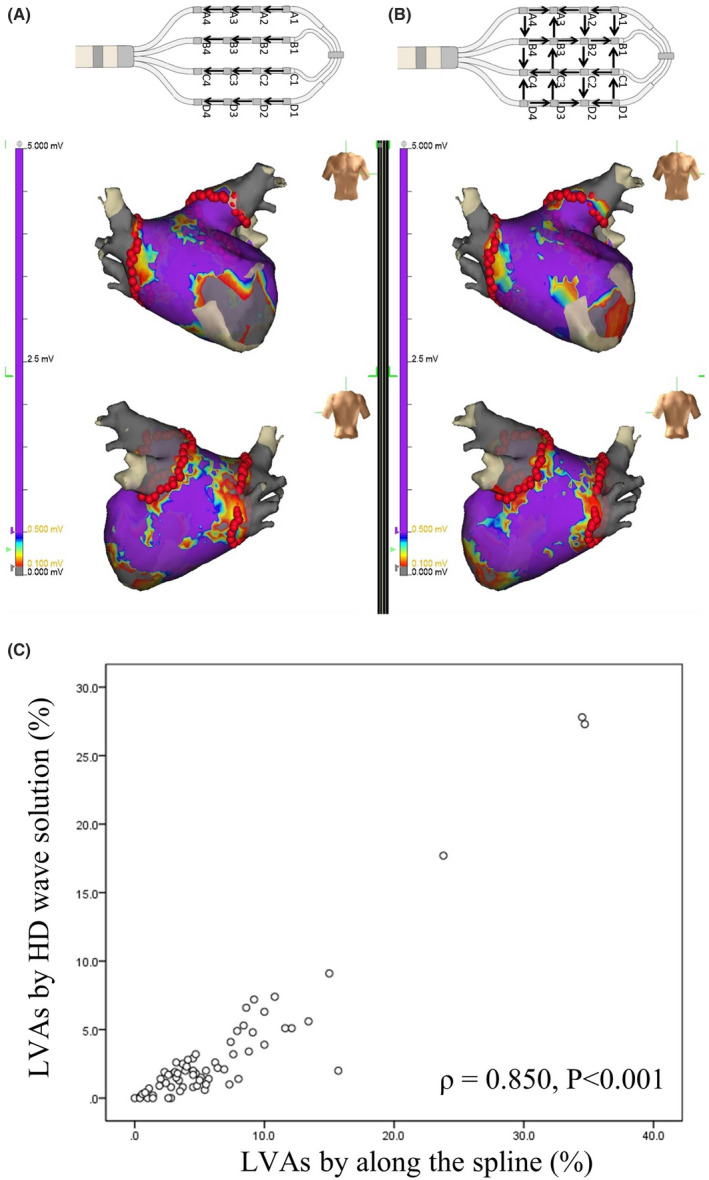
Voltage maps created using the two HDG bipolar configurations; along the spline (A) and HD wave solution (B), and the relationship to the LVA/LA surface between that using the two HDG bipolar configurations (C). The low‐voltage area (LVA)/left atrial (LA) surface was 20.5% (A) and 14.3% (B), respectively. The HDG using the HD wave solution could contribute to the bipolar recording regardless of the direction of the activation. The red tags show the PVI ablation points. The color coding was defined as follows: <0.1 mV = scar (gray), 0.1 to 0.5 mV = diseased atrial tissue, and >0.5 mV = healthy atrial myocardium (purple). (C) A tight relationship with the LVA/LA surface between that using the two HDG bipolar configurations was documented (Spearman correlation rho = 0.850, *p *< 0.001)

Two different voltage maps were created using the two HDG bipolar configurations (along the spline, which was equivalent to conventional mapping, and the HD wave solution). There was a significant difference in the LVA/LA surface between that using the HD wave solution and that along the spline (mean 3.3 cm^2^ vs. 6.5 cm^2^, *p* < .001). However, a tight relationship with the LVA/LA surface between that using the two HDG bipolar configurations was documented (Spearman correlation rho = 0.812, *p* < .001) (Figure 2). According to previous reports,^18^, ^19^ the extent of the LVZ (the HD wave solution vs. along the spline wave solution) was calculated as the percentage of the LA surface area and categorized into stages 1 (*n* = 54 vs. 36; minimum LVA, <5%), 2 (*n* = 10 vs. 27; mild, ≥5% to <20%), 3 (*n* = 2 vs. 1; moderate, ≥20% to <30%), and 4 (*n* = 0 vs. 2; extensive, ≥30%). There were five cases in which LVAs disappeared due to the HDG (HD wave solution). Sixty‐four of 66 (97.0%) patients with AF had minimum to mild LVAs (<20%) using the HDG with the HD wave solution regardless of an enlarged LAD and LA volume.

**FIGURE 3:**
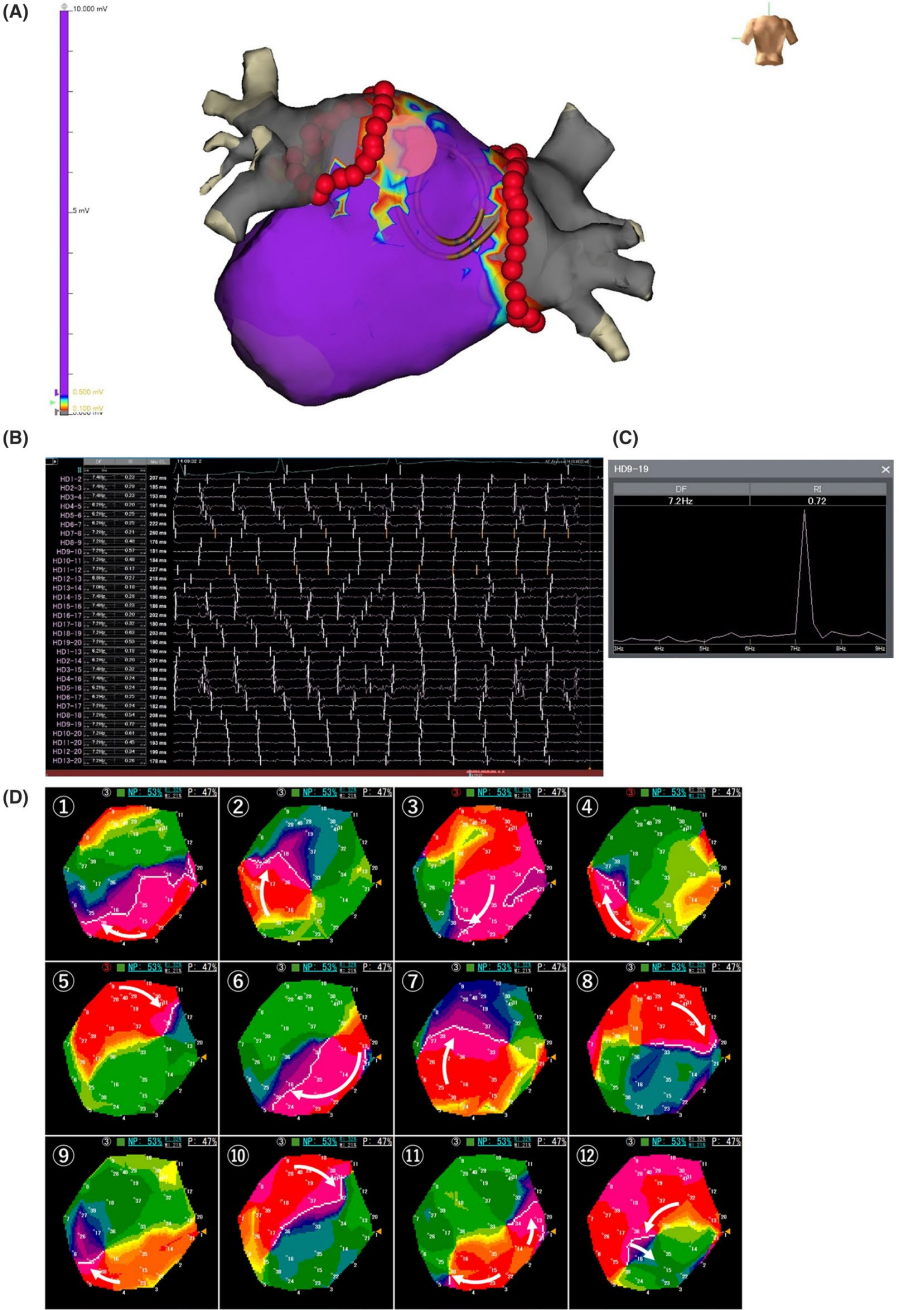
(A) A voltage map in the left atrium (LA) after the PVI. Minimal low‐voltage areas (LVAs) (LVAs/LA: 2.3%) were found in the posterior LA. The red tags show the PVI ablation points. (B) The spectrum of the high‐DF sites overlapping with LVAs in the posterior LA (7.2 Hz with RI 0.72) (pink tag). (C) Electrograms in the posterior LA after the PVI. Intra‐atrial bipolar electrograms recorded by a 20‐pole spiral‐shaped catheter during atrial fibrillation (AF) are shown. The mean AF cycle length is 148 ms. (D) ExTRa Mapping of the posterior LA after the PVI. The activation sequences during 720 ms of data (60‐ms × 12 consecutive time windows) are shown. The white lines indicate the head of the wavefronts and white arrows the direction of the wavefronts. In this case, a wave front traveling in the posterior LA forms a rotor lasting for three rotations. The nonpassively activated ratio (%NP) was 53%

There was a significant difference in the LVA/LA surface area between the patients with and without recurrent AF/AT (*p* = .004) as shown in Table 2. In addition, though there was no significant difference in %NPs of ≧50% that overlapped with the LVAs between the two groups (*p* = .282), there was a significant difference in the high‐DF sites of ≧7 Hz that overlapped with the LVAs between the two groups (*p* = .024). Furthermore, the number of sites that overlapped with all (LVAs, high‐DF sites ≧7 Hz and %NPs ≧50%) included only five (0.7%) out of 759 sites (two posterior, one septal, one roof, and one LAA site). A representative case is shown in Figure 3.

### 3.5 Outcome of the catheter ablation

**FIGURE 4:**
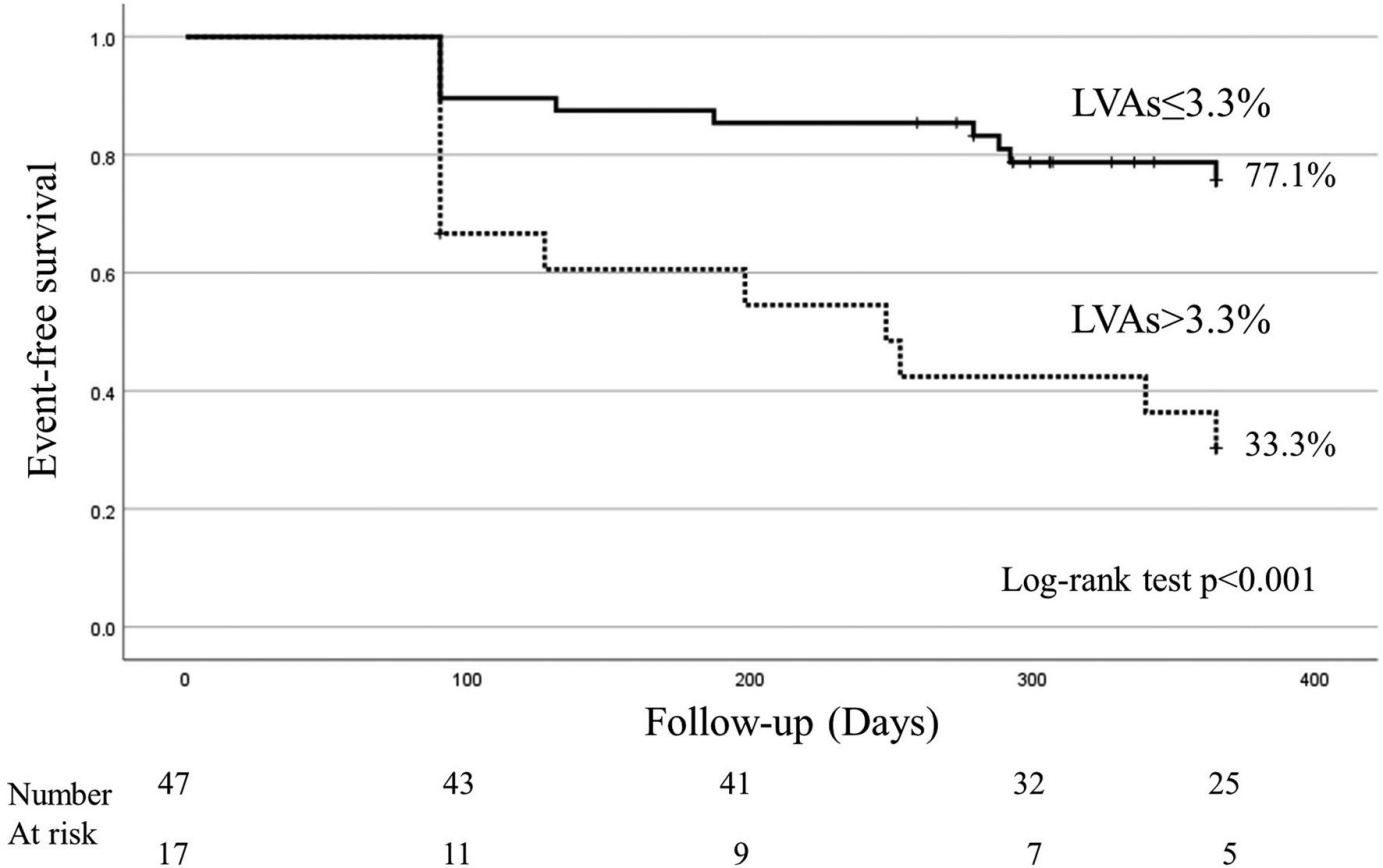
Kaplan‐Meier event‐free survival analysis for the cumulative freedom from AF/AT recurrence. AF/AT freedom was significantly greater in those with LVAs using the HDG of ≤3.3% than in those with LVAs of >3.3% after 1 procedure during 11.6 ± 0.8 months of follow‐up

A cutoff of 3.3% for the LVA/LA surface ratio was determined for the freedom from AF/AT recurrence with a 52.2% sensitivity and 88.4% specificity (area under the receiver operating characteristic curve of 0.657, *p* = .036). A Kaplan‐Meier event‐free survival analysis was conducted to assess the cumulative freedom from AF/AT recurrence. AF/AT freedom was significantly greater in those with LVAs of ≤3.3% than in those with LVAs of >3.3% after one procedure during 11.6 ± 0.8 months of follow‐up (77.1% vs. 33.3%, *p* < .001; Figure 4).

The number of patients receiving postablation AADs at the end of follow‐up after a blanking period was 13 (57%) among those with recurrent AF/AT. There were no cases of cerebral infarctions, cardiac tamponade, PV stenosis, or atrial‐esophageal fistulae.

### 3.6 Predictors of AF recurrence

As shown in Table 4, a univariate Cox proportional hazard regression analysis, including the AF duration, LA diameter, EF, LA volume, CHA2DS2‐VASc score, LVA/LA surface area after the PVI, max‐DF value, and max‐%NP value indicated that the LVA/LA surface area after the PVI (hazard ratio [HR] 1.079; confidence interval [CI], 1.025–1.135, *p* = .003) was significantly associated with AF/AT recurrence. In a multivariate analysis, the LVA/LA surface area after the PVI (HR 1.079; CI, 1.025–1.135, *p* = .003) was an independent predictor of AF recurrence.

**TABLE 4:** A univariate and multivariate Cox proportional hazard regression analysis

	Univariate			Multivariate		
Variable	HR	95% CI	*p* value	HR	95% CI	*p* value
AF duration	1.011	0.988–1.035	.359			
CHA2DS2‐VASc score	1.002	0.730–1.376	.990			
LA volume	1.010	0.995–1.024	.183			
LVAs/LA surface area	1.079	1.025–1.135	.003	1.079	1.025–1.135	.003
Max DF value	1.090	0.638–1.864	.752			
Max‐%NP	1.009	0.974–1.046	.616			

Abbreviations: %NP, nonpassively activated ratio; AF, atrial fibrillation; CI, confidence interval; DF, dominant frequency; HR, hazard ratio; LA, left atrium; LVAs, low‐voltage areas.

## 4 DISCUSSION

### 4.1 Major findings

The major findings of the present study were: (1) Though a relationship of the LVA/LA surface ratio between that using the two HDG bipolar configurations was documented, the HDG (HD wave solution) decreased the extent of the LVA. Therefore, most AF patients had minimum to mild LVAs using the HDG (HD wave solution) regardless of an enlarged LAD and LA volume. (2) There were no significant differences in the max and mean DF values and %NP after the PVI between the patients with and without recurrent AF/AT. (3) There was a significant difference in the LVA/LA surface area between the patients with and without recurrent atrial tachyarrhythmias. (4) The atrial tachyarrhythmia freedom was significantly greater in those with LVAs of ≤3.3% than in those with LVAs of >3.3% after one procedure during 11.6 ± 0.8 months of follow‐up. (5) In a multivariate analysis, the LVA/LA surface area after the PVI was an independent predictor of AF/AT recurrence.

### 4.2 Voltage mapping after a PVI

An increased amount of fibrosis as detected by LA voltage mapping has been shown to be a predictor of AF/AT recurrence after AF ablation.^5^, ^7^ The extent of the LVAs was categorized on the basis of the LA fibrosis grade evaluated by delayed‐enhancement magnetic resonance imaging (MRI).^18^, ^19^ However, the definition of the LVAs and their correlation with histological fibrosis remains controversial, because the bipolar voltage amplitudes depend on the electrode orientation relative to the direction of the wave front, electrode length, interelectrode spacing, and tissue contact.^20^ Furthermore, the HDG (HD wave solution) when used for bipolar recording can record not only the parallel but also the perpendicular activation to the splines, which differs from conventional mapping.^21^ Therefore, the HDG could create high‐density maps to define anatomical substrates regardless of the direction of the activation. In this study, the HDG (HD wave solution) decreased the extent of the LVA. The HDG may improve the directional sensitivity and exclude any false low voltages, and can detect the AF substrate more accurately, which would lead to avoiding any excessive ablation.

### 4.3 Frequencies and rotor mapping after a PVI

Atrial sites that represent local electrograms with high‐DFs may be associated with AF maintenance.^9^, ^13^ In addition, Sakata et al^17^ demonstrated that real AF drivers are contained in nonpassively‐activated areas where rotors and/or multiple wavelets are most frequently observed during a short recording time (5 s) with high reproducibility of the %NP. In the previous report, the highest DF and rotor positions were robust markers of the driver location during AF using a computational study.^22^ However, in this study, there were no significant differences in the DFs and %NP value by functional mapping after the PVI between the patients with and without recurrent AF/AT. Therefore, high‐DFs and rotors alone might not reflect the mechanism of recurrence of AF/AT in nonparoxysmal AF patients with atrial remodeling.

### 4.4 The sites overlapping with LVAs

In the previous study, 77% of the high‐DF sites overlapped with LVAs in the LA using conventional mapping catheters,^16^ and the overlapping sites using the HDG (HD wave solution) were decreased to 12% (20 sites out of 171) in this study. However, there was a significant difference in the high‐DF sites overlapping with LVAs between the patients with and without recurrent AF/AT. The HDG might have excluded false LVAs, which would help detect the AF substrate more accurately. Furthermore, there was no significant difference in the %NP ≧50% sites between the two because the %NP might indicate the frequency of the presence of rotors within a relatively large mapping area (diameter of 2.5 cm) as compared to the area overlapping with LVAs.

The selection of high‐DF sites overlapping with LVAs, not high‐DF sites alone, as targets may correct the shortcomings of an ablation based on the DF, as described previously.^14^, ^23^ The drivers are harbored within/in the vicinity of the LVAs, and fractionated activity, rotational activity, and discrete rapid local activity during AF in LVAs may contribute to the formation of high‐DFs.^6^ In this study, a combination of DF and LVA mapping could detect the critical selective atrial substrate necessary to maintain AF.

### 4.5 Study limitations

The present study was limited in several ways. First, this was a single‐center nonrandomized observational study with a relatively small population. Therefore, a selection bias of the clinical variables and different atrial substrates among the groups might have existed. Second, missed brief or silent AF episodes may have been underestimated in the present study because of noncontinuous monitoring during the follow‐up. Third, LVA mapping in the RA could not be performed due to the long procedure. Fourth, there may have been the possibility that the voltage and phase mapping may not have been adequately performed with sufficient catheter contact with the atrial tissue because we did not use a contact force sensing catheter for the mapping. Finally, we could not evaluate the efficacy of the ablation of the high‐DF sites that overlapped with LVAs.

## 5 CONCLUSIONS

Most AF patients had only minimum to mild LVAs using the HDG regardless of an enlarged LAD and LA volume in nonparoxysmal AF patients. The predictor of atrial tachyarrhythmia recurrence after the PVI was LVAs rather than DFs and rotors in nonparoxysmal AF patients.

## CONFLICT OF INTEREST

All the authors declare no conflict of interest related to this study.
